# What can we learn from pathophysiology and therapeutic targetable pathways from all genetic causes and associations in PH?

**DOI:** 10.1016/j.ijcchd.2024.100523

**Published:** 2024-06-27

**Authors:** Angela Balistrieri, Eckart De Bie, Mark Toshner

**Affiliations:** VPD Heart and Lung Research Institute, University of Cambridge, United Kingdom

**Keywords:** Genetics, PH

## Abstract

Pulmonary hypertension (PH) encompasses a group of conditions which ultimately lead to elevated pulmonary arterial pressure. PH is classified into five subgroups, of which Group 1 pulmonary arterial hypertension (PAH), is the most extensively studied. Numerous causal genes have been identified in PAH, most notably germline mutations in bone morphogenetic protein receptor type 2 (*BMPR2*) and the wider BMP pathway. Often when considering the genetics of PH, sporadic idiopathic and heritable PAH dominates the discussion. There are a number of reviews that elegantly describe the ‘state of the art’ in respect to group 1 PAH, however this focus misses the wider context of genetic conditions where PH is a feature, but outside of the framework of classical ‘idiopathic or heritable’ PAH. In addition to variants in genes within the TGF-β/BMP signaling pathway, genes which regulate ion channels, the extracellular matrix, inflammation, angiogenesis, and mitochondrial dysfunction have been shown to play a significant role in PH pathogenesis across different PH groups. In this review, we aim to cast the net wider to understand what we can learn from the spectrum of genetic conditions where PH is an acknowledged feature or complication, and what this tells us about the important cellular, molecular and systems physiology features that predispose to PH and consequently might be treatment targets.

## Funding sources

10.13039/100024877AB is funded by a Herchel Smith Postgraduate Scholarship, 10.13039/501100001446EDB is supported by the 10.13039/501100005370Gates Cambridge Trust, United Kingdom (Gates Grant number: OPP1144), MT is supported by the NIHR Cambridge Biomedical Research Centre

## 1 Introduction

Pulmonary hypertension (PH) is the consequence of a spectrum of diseases characterized by high pulmonary artery pressures currently clinically defined as a mean pulmonary arterial pressure of greater than 20 mmHg and pulmonary vascular resistance of greater than 2 Woods units [1]. Current imprecise estimates suggest that up to 1 % of the world's population may have PH, with rates and mortality highest in adults over the age of 65 [2]. To date, most forms of the disease are incurable, and PH is associated with significant mortality.

**Fig. 1:**
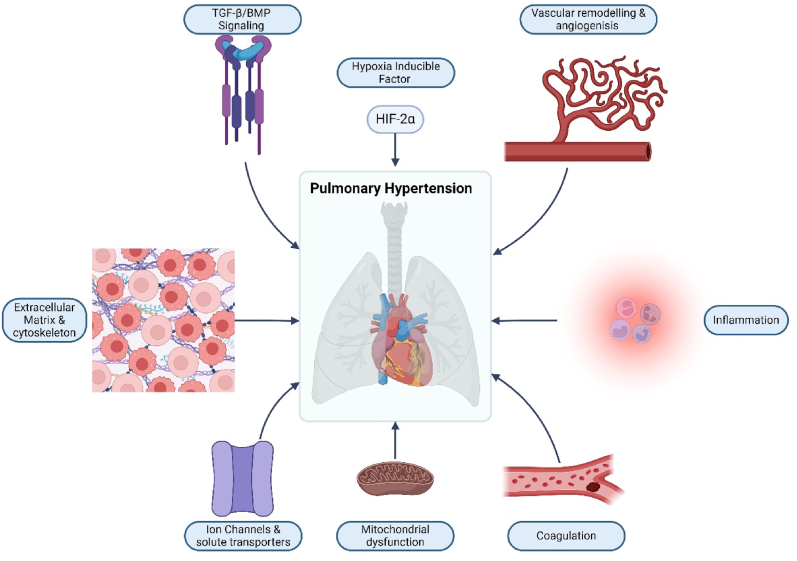
Summary of the cellular processes with mutations known to influence Pulmonary Hypertension.

PH is categorised into five World Health Organization (WHO) groups: (1) pulmonary arterial hypertension (PAH/group 1 PH), (2) PH due to left heart disease (group 2 PH), (3) PH due to lung disease and/or hypoxia (group 3 PH), (4) chronic thromboembolic PH and PH caused by pulmonary artery obstructions (CTEPH/group 4 PH), and (5) PH due to unknown causes (group 5 PH). Genetics involved in PH have been extensively studied in Group 1 heritable PAH (HPAH), with mutations in bone morphogenetic protein receptor type 2 (BMPR2) being causal in the majority of HPAH cases [1]. However, the genetics involved in other forms of PH are not as well known or discussed (see Fig. 1). There are important pathobiological lessons to be learned from considering the broader range of genetic conditions which cause or are associated with PH. In this review, we will discuss the mutations associated with all types of PH to clarify key pathways and to discuss important cellular and physiological processes in the development and trajectory of disease. It is now well-established that using human genetic evidence for therapy targets significantly improves the chances of success-a point well illustrated by recent innovation in PAH where Sotatercept, the only successful non-vasodilator therapy to make it to licensing, arose from targeting TGF signaling. A better understanding of what is already known about the common and unique genetic features associated with PH will help guide therapeutic innovation.

### 1.1 TGF-β/BMP signalling

The significance of abnormal transforming growth factor β (TGF-β) signaling has been extensively documented in the development of PAH. The majority of heritable PAH cases are caused by heterozygous germline mutations in the bone morphogenetic protein type II receptor (*BMPR2*) [1]. However, these mutations are inherited with incomplete penetrance at just 10–30 %, suggesting a “second hit” of other genetic, epigenetic, or environmental factors are needed in order for the disease to manifest [1]. In addition to mutations in *BMPR2*, other mutations within the TGF-β superfamily have been implicated in the development of PAH. A recent systematic review ranked the relative strength of evidence supporting different PAH-related mutations, and listed several mutations in the TGF-β signaling pathway in addition to *BMPR2* which were classified as having definitive, moderate, limited and disputed evidence for being causal in PAH [3]. Those with definitive evidence are outlined in Table 1. Six genes were described as having limited evidence *(AQP1, BMP10, FBLN2, KLF2, KLK1,* and *PDGFD),* and 5 genes *(BMPR1A, BMPR1B, NOTCH3, SMAD1,* and *SMAD4)* were disputed because of a lack of evidence. What is notable about the list of genes that are definitely causal and possibly causal is the strong involvement of the BMPR2 pathway and the preponderance of genes known to be involved in pulmonary vasculogenesis or angiogenesis. Perhaps unsurprisingly, a heritable defect in pulmonary vascular development, would seem to underlie a significant proportion of cases, though this does not rule out additional contribution of these genes and pathways to other processes such as ongoing vascular homeostasis, inflammation, proliferation etc.

**Table 1:** Genes with strong evidence of causal association in idiopathic/heritable PAH.

Gene	PH phenotypic association	Putative genetic consequence	Potential distinguishing clinical and examination features
*BMPR2*	Heritable and idiopathic PAH	Haploinsufficiency	No specific or diagnostic clinical features described
*ATP13A3*		Unknown	
	
*KCKN3*		Haploinsufficiency	
		Haploinsufficiency	
*SMAD9*		Haploinsufficiency	
*SOX17*	Heritable and idiopathic PAH, Congenital heart disease	Unknown	
*CAV1*	Heritable and idiopathic PAH, Lipodystrophy	Gain of function; dominant negative	deficiency of subcutaneous adipose tissue
*TBX4*	Heritable and idiopathic PAH/small patella syndrome (ischiopatellar dysplasia)/parenchymal lung disease/bronchopulmonary dysplasia Persistent PH of the neonate	Unknown	Patellar aplasia Skeletal abnormalities- in particular attention to pelvis, knees and feet
*EIF2AK4*	Pulmonary veno-occlusive disease/pulmonary capillary haemangiomatosis	Loss of function	Distal phalangeal clubbing
*KDR*	Heritable and idiopathic PAH	Loss of function	
*ENG*	Heritable and idiopathic PAH/hereditary haemorrhagic telangiectasia	Unknown	Telangiectasia Abnormal blood vessel formation Visceral arteriovenous malformations Bleeding diathesis
*ACVRL1*		Haploinsufficiency	
*GDF2*		Haploinsufficiency	

In addition to being implicated in Group 1 adult PAH, several genes in the TGF-β superfamily have also been shown to play a role in other forms of PH. Germline mutations in *BMPR2, SMAD9, ENG, BMP10, CAV1, SOX17*, and *TBX4* were identified as risk variants in childhood PAH with some being associated with congenital heart disease [4]. Dysregulated TGF-β signaling has also been implicated in other PH groups [5]. The power of leveraging human genetic data in drug target identification has received an important validation in the recent demonstration of surprisingly large functional treatment effect sizes in diverse populations of group 1 PAH patients already established on dual and triple therapy and targeting the TGF-β superfamily with an activin receptor Fc fusion protein (Sotatercept) [6]. This work has proven the central importance of the pathway in diseases not restricted to the genetic forms and represents a genuine step change in our therapeutic armamentarium and it is our expectation that there will be backwards translation and refinement of the mechanism of action that will further our understanding of disease.

### 1.2 Ion channels and solute transporters

It is often overlooked that most of the existing therapies ultimately target cellular ionic function with intracellular calcium signaling sitting downstream of all the upstream drug targets. Calcium, potassium, sodium, and chloride, play an important role in the development of PH. Potassium ion (K^+^) channels are transmembrane proteins which regulate resting membrane potential. Downregulation or loss-of-function mutations of K^+^ channels leads to membrane depolarization, activating voltage-gated Ca^2+^ channels, and increasing intracellular Ca^2+^ concentration, which ultimately increases PASMC proliferation and constriction [7].

In Group 1 PAH, two genes regulating potassium ion levels have shown strong evidence for playing a causal role in disease development: *potassium channel subfamily K member 3 (KCNK3*) [[8], [9]] and *ATP-binding cassette subfamily C member 8 (ABCC8)*. *KCNK3*, or *TASK-1*, encodes a member of the K2P channel family, is expressed in a wide variety of cell types including PAECs, PASMCs, and the right ventricle [7]. Genetic variants in *KCNA5* have also been found in patients with PAH, as well as reduced expression and activity of Kv1.5, encoded by *KCNA5*, however the exact mechanism is still relatively unknown [[8], [9]]. *ATP-binding cassette subfamily C member 8 (ABCC8)* encodes for sulfonylurea receptor-1 (SUR1), which is the regulatory subunit of the ATP sensitive potassium (K_ATP_) channel. Reduced channel function due to heterozygous mutations have been shown to potentially play a role in the development of HPAH and Idiopathic PAH (IPAH) [1], PAH associated with congenital heart disease (APAH-CHD) [6], and PAH associated with connective tissue disease (APAH-CTD) [10].

*ATP13A3* is another channelopathy gene that has been validated in PAH cohorts [3,11]. *ATP13A3* regulates cell proliferation and growth by encoding a transmembrane polyamine transporter. Loss of function mutations of *ATP13A3* lead to endothelial cell damage through impaired polyamine homeostasis, reduced proliferation, increased permeability, and increased apoptosis [12].

### 1.3 Extracellular matrix and cytoskeleton

Aberrant extracellular matrix (ECM) deposition and remodeling is a key feature of PH, and increased fibrosis is observed in the intima, media, and adventitia of the vessel wall. A number of genes which regulate different parts of the ECM have been implicated in different PH groups. Mutations in *FBLN2,* a glycoprotein localized in the basement membrane of the extracellular matrix and involved in matrix regulation, have been shown to play a causal role in Group 1 PAH [13]. Genes involved in other diseases which have a high prevalence of PH have also been shown to regulate the ECM. For instance, patients with combined pulmonary fibrosis and emphysema (CPFE) are at a greater risk for developing PH, with CPFE presenting frequently at approximately 50 % with PH [14]. Matrix metallopeptidase 1 (*MMP-1*) regulates extracellular matrix and collagen degradation, and its upregulation has been implicated in idiopathic pulmonary fibrosis and in CPFE [15]. Polymorphisms of this gene, combined with environmental factors such as smoking, can cause upregulation of collagen degradation and increase risk of developing CPFE and PH. MMP-1 levels are increased in the lungs, serum, and M1-polarized macrophages of PAH patients, potentially due to dysregulated MAPK signaling upstream of MMP-1 expression [16]. Although the exact mechanism by which MMP-1 contributes to CPFE is not yet well understood, current findings suggest a potential link between MMP dysfunction and pathological vessel remodeling in PH.

### 1.4 Inflammation

Chronic inflammation contributes to vascular remodeling and PH, as recruitment and accumulation of perivascular inflammatory cells is observed in PH. From a genetics standpoint, several variants which influence inflammation have been directly and indirectly tied to PH. The largest international GWAS in idiopathic PAH identified novel genetic variants in a loci associated with human leukocyte antigen DPA1 *(HLA-DPA1)*/*HLA-DPB1* [17]. *HLA-DPA1* is a member of the HLA gene family, which encode major histocompatibility complexes Class I and Class II and play an important role in the adaptive immune system and in antigen presentation to immune cells [17]. Systemic lupus erythematosus-PAH has reported variants associated with HLA-DQα1 [18]. A recent GWAS study also noted variance in an *HLA-DRA* loci as significantly associated with CTEPH [19]. Although the mechanism of HLA genes in causing PAH is not clear, their role in MHC/peptide-CD4^+^ T cell receptor affinity implicates dysregulated inflammation as having a causal role in PAH pathogenesis across differing classes of disease.

The JAK/STAT signalling pathway is thought to be important in inflammation and vascular remodeling in addition to roles in haemostasis and coagulation. For example, in the context of polycythemia rubra vera there is an association with the JAK2V617F *polymorphism*. This has been suggested to associate with higher rates of PH, though determined on echocardiography only suggested to be high though as assessed by echo [19]. This is in the context of small cross-sectional studies with likely high bias. Although *JAK2* mutations have been suggested to be more common in PH patients compared to healthy controls, this has not been confirmed in large scale international cohorts looking at both common and rare variation [11,17]. Recently, a case study reported a patient who had a loss-of-function mutation in the *AIRE* gene, which regulates autoreactive T-cell clearance and production of regulatory T cells, developed PAH, supporting the connection between regulatory T cells and PAH pathogenesis [19].

### 1.5 Hypoxia inducible factor (HIF)

Hypoxia inducible factor (HIF), a heterodimeric transcription factor, regulates oxygen homeostasis and is believed to play a critical role in the pathogenesis of PH. Hypoxic conditions activate HIF, leading to a broad range of effects on genes which regulate angiogenesis, vascular tone, proliferation, and survival. Dysregulation in HIF signaling has been shown to be involved in various forms of PH, including PAH or group 1 PH, PH due to lung disease or hypoxia, group 3 PH, and COPD [20]. While many factors can influence HIF signalling, such as inflammation, hypoxia, and mechanical stretch, genetic predispositions could play an important role in dysregulated HIF signaling.

Genetic variations of the HIF pathway have been observed in Chuvash polycythemia, a disorder characterized by loss of function mutations in the von Hippel Lindau (*VHL*) gene [20]. Patients with Chuvash polycythemia are at a higher risk of developing PH (group 5 PH), have an increased pulmonary arterial pressure at baseline, and increased respiratory rates [20].

Genome wide selection studies on high altitude populations demonstrate that genetic variants in the HIF pathway are positively selected for adaptation to lower oxygen conditions, Tibetans are enriched for variants in *EPAS1* (HIF-2α) and shown to have lower pulmonary arterial pressure (PAP) and decreased pulmonary vasoconstriction under hypoxic conditions [21]. Andean populations there is polymorphism in the *PHD2* gene, a major intracellular stabilizing protein for HIFs and Tibetans with *EGLN1* and *EPAS1* variants have decreased hypoxic pulmonary vasoconstriction even at low altitudes [20]. Inhibition of HIF is an obvious therapeutic target that may be relevant to several different PH classes, especially where hypoxia is prominent.

### 1.6 Vascular remodeling and angiogenesis

One of the hallmarks of PAH is lung vessel obliteration due to dysregulated angiogenesis. The increasing list of genes that cause heritable and idiopathic PAH has confirmed the importance of developmental pathways relevant to and expressed in the pulmonary vasculature and not restricted BMPR2 and its upstream and downstream pathway. Vascular endothelial growth factor (VEGF) is a proangiogenic mitogen in the vascular endothelium and is a downstream target of HIF. Increased VEGF-A and VEGFR2 expression has been well documented in PH and Mutations in *KDR* (kinase insert domain receptor*)*, which encodes VEGFR2, have been associated with HPAH notable for an association with low carbon monoxide diffusing capacity [1].

Rare variants in SRY-box transcription factor 17 (*SOX17)* have also been strongly associated with PAH [11]. SOX17 is a proangiogenic transcription factor and promotes expression of VEGFR2, as well as being involved in development of the endoderm, cardiomyocytes, vascular endothelium, and hemapoietic cells.

### 1.7 Coagulation/haemostasis

Polymorphisms in genes which play a role in coagulation have been shown to be significantly associated with CTEPH in a multinational GWAS of 1907 cases of CTEPH; *FGG* and *ABO* polymorphisms were the most significant associations, followed by other coagulation related genes including *F2* and *F11* [19]. The *FGG* gene encodes for the fibrinogen γ chain, a subunit of fibrinogen and a major clot component. The same study found no evidence of genetic overlap between IPAH and CTEPH, and that CTEPH had more genetic similarity to acute pulmonary embolism (PE). Though CTEPH and IPAH share pathophysiological features, the genetics of disease demonstrate that they have different underlying mechanisms.

Mitochondrial Dysfunction.

Mitochondrial dysfunction and resultant oxidative stress may be a common dysregulated process across diverse causes of PH [21]. Due to their importance in regulating ATP production, proliferation, apoptosis, and reactive oxygen species (ROS) production, mitochondrial dysregulation may associate with PH through a number of different mechanisms. Mitochondria act as oxygen sensors in order to determine when to make a metabolic “switch” between aerobic and anaerobic pathways depending on oxygen availability [22]. Normally, hypoxic conditions would lead to an increase in anaerobic respiration, however in PH this switch can occur in normoxia. Termed the Warburg effect, this aerobic glycolysis has been observed in the PAECs and PASMCs of IPAH patients, as well as PH animal models [22]. This effect not only leads to less efficient energy production, but is believed to contribute to the anti-apoptotic phenotype observed in disease [22].

Knowledge of the genetics driving mitochondrial dysfunction in PH is limited, however several genetic variants related to mitochondrial function have been implicated. Deficiency and mutations in iron-sulfur (Fe–S) biogenesis genes, iron-sulfur duster assembly protein (*ISCU1/2*), BolA family member 3 (*BOLA3*) and Transmembrane protein 70 (TMEM70) [23,24]. A homozygous variant in *COX5A*, a subunit of the mitochondrial respiratory chain complex IV, was reported in a case of siblings with PAH and mitochondrial complex IV gene [25] and *COX7B* in a paediatric patient [26]. Both suggest dysfunctional complex IV biogenesis is linked to disease development. Mutations in the *SARS2* gene, which encodes the mitochondrial seryl-tRNA synthetase, is believed to lead to the rare mitochondrial disease, HUPRA syndrome [27]. HUPRA syndrome, or hyperuricemia, PH, and renal failure syndrome, is an autosomal recessive genetic disease caused by loss of function seryl-tRNA synthetase. This subsequently inhibits tRNA synthesis resulting in dysregulation of the oxidative phosphorylation system.

### 1.8 Other metabolic and miscellaneous disorders

A variety of other rare diseases in less classifiable ontologies are associated with PH. Without extensively covering reported cases, they include Gaucher's disease, a lysosomal storage disorder associated with gene encoding acid beta-glucosidase (*GBA*) mutations leading to a reduction in the activity of acid β-glucosidase and accumulation of glycolipid [28], RASopathies-developmental disorders caused by a mutation in the Ras and associated Mitogen-Activated Protein (MAP) kinase signaling pathways [29] and the Metabolism Of Cobalamin Associated C gene (*MMACHC*) where case reports have demonstrated reversibility of PH with parenteral hydroxocobalamin treatment [30]. Drawing direct lines of pathobiological underpinning causation in some of these rare diseases and PH can be challenging.

### 1.9 Gene therapy in PH

Due to the association of numerous genetic mutations with the aetiology of PH and recent technological advancements in the field, gene-therapy could be a promising method for the treatment of PH. Non-viral and viral delivery systems have shown promise in preclinical data *in vitro* and *in vivo* in animal models thus far [31]. However poor transfection efficiency and reproducibility, along with cell cytotoxicity present challenges in utilizing these techniques [31]. To date, adeno-associated vectors (AAVs) have shown the most promise as a potential gene therapy in clinical trials outside of PH [32]. Overall, despite *in vitro* efficacy, gene therapy in PH has several obstacles to overcome in order be applicable in a clinical setting, such as limited duration of action, target organ delivery and engagement. More research is needed to further develop these systems to be applied in a patient setting.

## 2 Summary

PH encompasses a wide range of pathologies with varying etiologies and causal mechanisms. Despite their distinguishing features, there are common cellular processes which seem to be pathological across the varying PH group. The genetics of PH have given us a better understanding of the central importance of pathways that are specifically involved in pulmonary vascular development and homeostasis, leading to the first licensed treatment that doesn't rely on vasodilation. As in other diseases, the genetics of PH more broadly are a powerful tool for understanding pathobiology and for drug target selection. Dysregulated inflammation, extracellular matrix deposition, HIF signaling and mitochondrial dysfunction as well as several other potential mechanisms should arguably be prioritized in drug development, and we need to think more widely about the phenotypes that may be tractable to modulating these processes. Understanding the genetics of PH sheds light on PH pathogenesis and potential therapeutic targets.

## CRediT authorship contribution statement

**Angela Balistrieri:** Writing – review & editing, Writing – original draft, Methodology, Formal analysis, Data curation, Conceptualization. **Eckart De Bie:** Writing – review & editing, Methodology, Data curation, Conceptualization. **Mark Toshner:** Writing – review & editing, Writing – original draft, Formal analysis, Conceptualization.

## Declaration of competing interest

The authors declare the following financial interests/personal relationships which may be considered as potential competing interests:Mark Toshner reports a relationship with Janssen Pharmaceuticals Inc that includes: consulting or advisory. Mark Toshner reports a relationship with Merck Sharp & Dohme UK Ltd that includes: consulting or advisory. Mark Toshner reports a relationship with Apollo Therapeutics that includes: consulting or advisory. If there are other authors, they declare that they have no known competing financial interests or personal relationships that could have appeared to influence the work reported in this paper.
